# Prevalence of childhood disability and the characteristics and circumstances of disabled children in the UK: secondary analysis of the Family Resources Survey

**DOI:** 10.1186/1471-2431-10-21

**Published:** 2010-04-16

**Authors:** Clare M Blackburn, Nick J Spencer, Janet M Read

**Affiliations:** 1 School of Health and Social Studies, University of Warwick, Coventry CV4 7AL, UK

## Abstract

**Background:**

Robust data on the prevalence of childhood disability and the circumstances and characteristics of disabled children is crucial to understanding the relationship between impairment and social disadvantage. It is also crucial for public policy development aimed at reducing the prevalence of childhood disability and providing appropriate and timely service provision. This paper reports prevalence rates for childhood disability in the United Kingdom (UK) and describes the social and household circumstances of disabled children, comparing these where appropriate to those of non-disabled children.

**Methods:**

Data were generated from secondary analysis of the Family Resources Survey, a national UK cross-sectional survey, (2004/5) which had data on 16,012 children aged 0-18 years. Children were defined as disabled if they met the Disability Discrimination Act (DDA) definition (1995 and 2005). Frequency distributions and cross-tabulations were run to establish prevalence estimates, and describe the circumstances of disabled children. To establish the association between individual social and material factors and childhood disability when other factors were controlled for, logistic regression models were fitted on the dependent variable 'DDA defined disability'.

**Results:**

7.3% (CI 6.9, 7.7) of UK children were reported by as disabled according to the DDA definition. Patterns of disability differed between sexes with boys having a higher rate overall and more likely than girls to experience difficulties with physical coordination; memory, concentration and learning; communication. Disabled children lived in different personal situations from their non-disabled counterparts, and were more likely to live with low-income, deprivation, debt and poor housing. This was particularly the case for disabled children from black/minority ethnic/mixed parentage groups and lone-parent households. Childhood disability was associated with lone parenthood and parental disability and these associations persisted when social disadvantage was controlled for.

**Conclusion:**

These analyses suggest that UK disabled children experience higher levels of poverty and personal and social disadvantage than other children. Further research is required to establish accurate prevalence estimates of childhood disability among different black and minority ethnic groups and to understand the associations between childhood disability and lone parenthood and the higher rates of sibling and parental disability in households with disabled children.

## Background

There is considerable global concern to reduce the prevalence of childhood disability and to improve health, social and educational outcomes in order to extend social participation for disabled children [1]. Both cases require reliable prevalence estimates of childhood disability and robust quantitative data on disabled children's characteristics and circumstances. Prevalence estimates vary considerable between and within nations, and in many countries data on disabled children is lacking [2,3]. This is also the case in the United Kingdom (UK). Although a range of UK administrative, population census and survey sources contain data on this important group, the availability of up-to-date, reliable quantitative data to inform public health planning and the commissioning and provision of services at national and local level has been very limited [4-8]. This paper aims to contribute to this information gap by reporting on a secondary analysis of a national, representative cross-sectional survey, the Family Resources Survey (FRS). It reports prevalence estimates of childhood disability for the total child population by age, sex, ethnicity and impairment type and examines the relationship between childhood disability and social circumstances.

Reliable quantitative data on disabled children has been lacking for a number of reasons. Defining and measuring childhood disability and the circumstances of disabled children and their households present a number of complex theoretical, philosophical and technical issues [9]. These affect both prevalence estimates and related socio-demographic information about the children and their households. The multi-dimensional, dynamic and contested nature of disability may make it inherently difficult to measure (for a detailed discussion of these issues, see [10]). Furthermore, the way that disability is defined determines both the type of data being collected and the process of data-collection. It has been argued that it is by no means obvious or certain who might be regarded or might regard themselves as rightfully being inside or outside the disability category [11]. In addition, the perception of disability as a fixed and distinct status has been called into question as has the related notion of a simple dichotomy between those who are disabled and those who are not [12-14]. In turn, definitions and understandings of disability inevitably shape the range of responses by research participants. The willingness of parents to identify their children as disabled, for example, may vary according to whether the definition used reflects their own definition of disability generally, their perception of any difficulties their child may experience and the implications as they understand them, of defining their child as 'disabled'.

In addition to the issues discussed above, gathering and providing information on disabled children and their households is further complicated by validity and reliability issues. Estimating the prevalence of childhood disability is subject to the same validity and reliability issues affecting the measurement of adult disability. These include the representativeness of the sample and the episodic nature of some disabilities [10]. In addition, there are a range of issues relating specifically to the measurement of disability in children. These include the relative rarity of disability in early childhood, the difficulty of ensuring the inclusion of all disabled children, including those who live away from home for some of the time, and the use of adult specific questions that fail to take account of the child's age and development,[15].

Despite inherent difficulties in defining and measuring childhood disability and the circumstances and characteristics of disabled children, such data is key to the development of appropriate and timely service provision for this group and their families.

A number of key UK government data sources now offer the opportunity to generate up-to-date prevalence estimates and other information on disabled children and their households but their data remain largely unpublished. This paper uses data from a key source, the FRS. The FRS's advantage over other government surveys is that it collects information on children who would be classed as disabled under the Disability Discrimination Act (DDA), 1995 and 2005 and therefore have rights under this legislation. Given the obligations owed to these children by a range of bodies, it is important to know how many there are and their characteristics and circumstances.

Data on the prevalence of child disability and the characteristics and circumstances of disabled children is key to understanding the relationship between impairment and restrictive social conditions, and to informing policy development that aims to both reduce childhood disability and provide appropriate and timely service provision for this group and their families.

In the UK, a significant programme of policy reform aims to improve outcomes for disabled children and their families. Public authorities, including those with public health responsibilities, have a range of existing duties in relation to disabled children and their families. These include improved service quality and capacity in health and social care; more choice and flexibility in the type of services and support provided; responsive and timely support; better support in the early years and at transition to adulthood; increased social inclusion [8,7,16,17,15]. These responsibilities are likely to be extended by the introduction of further equalities legislation, increasing the need for information on this group.

## Methods

Data presented here were generated from secondary analysis of the FRS (2004-5).

The FRS is considered by the Department of Work and Pensions to be its key source of information on disability [18]. It is an annual cross-sectional survey that collects information on the incomes and circumstances of approximately 29,000 private UK households which contain around 16,000 children age 0-18 years.

Details of the FRS survey design, sampling procedure and survey methods and instruments are available at http://research.dwp.gov.uk/asd/frs/2004_05/index.asp. A total of 28,041 private households fully cooperated in the 2004-5 survey, a response rate of 62%. The data presented in this paper are generated from the sub-sample of 16,012 children aged 0-18 years who lived in 8,711 households.

## Measures

### Child disability

**Figure 1:**
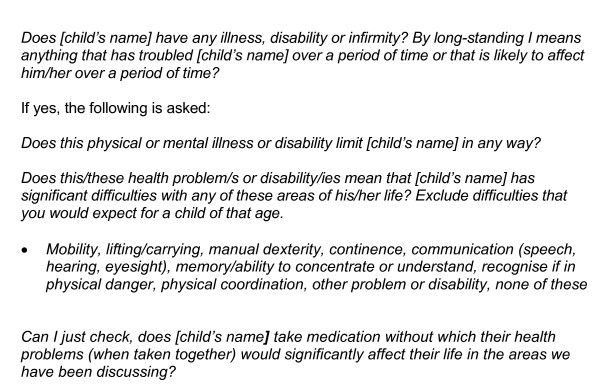
**FRS question sequence to identify children with a DDA-defined disability**.

children were defined as disabled if they met the DDA criteria (1995 and 2005) for a disabled person. The measure 'DDA-defined disability' includes children with a limiting longstanding (12 month duration or longer) illness, disability or infirmity experiencing one or more significant difficulties or health problems. It also includes those who would have such difficulties or problems if they did not take medication/s. The question sequence used to establish whether a child would be defined as experiencing a DDA-defined disability can be found in Figure 1.

### Social and household circumstance

Measures included the child's age (0-4, 5-11, 12-15, 16-18); sex; number of adults in the family unit (1 v 2); number of dependent children in the family unit (2 or less v 3 or more); number of adults with a DDA-defined disability in family unit (0 v 1 or more); housing tenure (owner occupied v rented/other). Due to the small number of children in Black and minority ethnic groups, ethnicity was dichotomised according to the reported ethnicity of the head of family (white UK/other v black/minority ethnic/mixed parentage).

### Income

A number of measures of income were included: household income quintiles and median household income. Income data represents equivalised net disposable income after housing costs have been taken into consideration. Equivalisation allows the living standards of households that vary in size and composition to be compared and is based on the common sense notion that a family with several people requires a higher income than a single person to have the same living standard [19]. Income was adjusted using the equivalisation variable available in the data set, the McClements equivalisation scales [19].

### Material deprivation

Measures were derived from a block of questions on parental reported perception of: whether they could afford a number of items they would like but cannot afford; ability to stay clear of debts. These items have been tested in other surveys (Families and Children Study, British Household Panel Survey and the Poverty and Social Exclusion Survey) and were included as they were items generally considered important to family living standards. We constructed a deprivation index from the child and household deprivation items available in the data set. A score of 1 was given if an item was considered wanted or needed but could not be afforded and the scores summed to give a total score for the number of items lacked.

## Data analysis

Frequency distributions were run to establish prevalence estimates. These data were weighted, using the grossing factors supplied for the FRS, to adjust for non-response and for population estimates for each of the home nations. All other analyses used non-weighted data. To examine differences in social and material circumstances between groups (disabled versus non-disabled children), the Pearson Chi-square test was reported except for two-by two tables when the Yates' continuity correction was used. As income data was not normally distributed, the median value was recorded and the Mann-Whitney U test used to compare differences in equivalised median income.

In order to examine the associations between individual, social and material factors when other variables were controlled for, logistic regression models were fitted on the dependent variable 'DDA defined disability'.

Factors identified as statistically significant in the bivariate analyses were entered into a direct logistic regression analysis to produce odds ratios with 95% confidence intervals. Where more than one socio-economic indicator was statistically significant in the bivariate analysis, the indicator with the greatest odds (housing tenure) was entered into the model in order to avoid multicollinearity. All the remaining demographic factors were entered into the multivariate direct logistic regression analyses. The number of dependent children in the family unit was not significant in the bivariate analysis but was included in the multivariate analysis as it is has been shown to be an important determinant of poverty in households with children [20]. The odds ratios indicate how much the odds of the child having a DDA defined disability are increased by being in a more disadvantaged group. Differences between groups were considered significant at the 5% level (P = 0.05). Although the FRS has a complex sample design, it was not possible to control for the effect of clustering in the analyses as the variable for identifying the primary sampling unit (postcode address file) was not available in the publicly available dataset. As a result, the standard error of the odds ratios are likely to be under-estimated in the analysis reported here leading to narrower confidence intervals.

## Results

### Estimates of childhood disability

The final sample consisted of 16,012 children age 0-18 years. Of these, 7.3% (CI 6.9, 7.7) were reported as experiencing a DDA-defined disability. Nationally, this amounts to 952,741 children. Table 1 gives UK prevalence estimates for children with a DDA-defined disability by age, sex, and ethnicity. The prevalence of reported DDA-defined disability was higher among boys than girls and lowest among children aged 0-4 years. Among boys and girls, prevalence increases until age 12-15 years, after which it falls slightly.

**Table 1:** Child population prevalence estimates for DDA-defined disability, by sex, age and ethnic group, UK, 2004/5

	n	% [95% confidence intervals]
**All children**	952,741	7.3 [6.9. 7.7]

**Sex**		
Boys	583,278	8.8 [8.2, 9.4]
Girls	369,463	5.8 [5.3, 6.3]

**Age**		
0-4 years	129,074	3.7 [3.2, 4.3]
5-11 years	409,862	8.2 [7.6, 8.9]
12-15 years	302,485	9.5 [8.6, 10.5]
16-18 years	111,320	8.5 [7.2, 10.0]

**Ethnicit***y*		
White UK/other	870,603	7.6 [7.2, 8.0]
Mixed parentage	12,186	9.5 [5.4, 14.7]
Indian	7,947	2.7 [1.4, 5.4]
Pakistani and Bangladeshi	24,097	5.1 [3.4, 7.6]
Black or Black British	26,610	7.1 [5.1, 9.9]
Other ethnic group	11,298	4.4 [3.6, 7.2]

Table 2 shows the proportions of children with a DDA-defined disability reported as experiencing substantial difficulties with specific areas of daily living. The most commonly reported difficulties were with memory, ability to concentrate and/or learn and with communication. Reported difficulties with memory, concentration and/or learning, with communication and with physical coordination, were more commonly reported in boys than girls. The category 'DDA-defined disability', in addition to including children who experienced substantial difficulties with daily living, also included those who took medication without which their health problems would result in significant difficulties in daily living. In total, 1.9% of all children and 25% of children with a DDA-defined disability fell in to this category.

**Table 2:** Proportions of children with a DDA-defined disability reported as experiencing particular difficulties FRS, 2004/5

	% [95% confidence interval] of population (weighted)		% [95% confidence intervals] of disabled children (non-weighted)				
**Difficulty/problem experienced**	**All**		**Male**		**Female**		**p**

Mobility	193,950	1.5 [1.3, 1.7]	150	20.7 [17.9, 23.8]	98	21.1 [17.8,25.3]	0.940

Lifting and carrying	84,759	0.7 [0.6, 0.8]	66	9.1 [7.2, 11.4]	44	9.5 [7.1, 12.5]	0.921

Manual dexterity	107,798	0.8 [0.7, 1.0]	93	12.8 [10.6,15.5]	41	8.8 [6.6, 11.7]	0.040

Continence	88,748	0.7 [0.6, 0.8]	66	9.1 [7.2, 11.4]	48	10.3 [7.9, 13.4]	0.556

Communication	255,534	2.0 [1.8, 2.2]	210	29.0 [25.8, 32.4]	106	22.8 [19.2, 26.8]	0.022

Memory, concentration, learning	288,203	2.2 [2.0, 2.4]	260	35.9 [32.5, 39.5]	96	20.6 [17.2, 24.6]	<0.001

Recognising physical danger	171,352	1.3 [1.1, 1.5]	154	21.3 [18.5, 24.4]	55	11.8 [9.2, 15.1]	<0.001

Physical coordination	167,585	1.3 [1.1, 1.5]	151	20.9 [18.1, 24.0]	64	13.8 [10.9, 17.2]	0.002

Other	268,427	2.1 [1.9, 2.3]	214	29.6 [26.4, 33.3]	135	29.0 [25.1, 33.3]	0.846

Difficulty if didn't take medication	247,898	1.9 [1.7, 2.1]	160	22.1 [19.2, 25.3]	141	30.0 [26.3, 34.7]	0.452

CI calculations performed on CI Calculator for single proportions at http://www.pedro.org.au/english/downloads/confidence-interval-calculator/ accessed 8.12.09

For some children, disability was complex with children experiencing difficulties in more than one area of daily living. A third of disabled children (35.2%) experienced two to four difficulties and 13.3% experienced difficulties in five or more areas of daily living.

### Family and living circumstances

Table 3 reports on the living circumstances of disabled children and compares these with those of non-disabled children. Almost two-thirds of disabled children lived in two-parent families. The proportion living in lone parents families however was significantly greater than that for non-disabled children. A quarter lived with one or more siblings who also had a DDA-defined disability. A further 0.2% lived with a disabled child/ren who lived in the same household but was not a sibling. Almost one-half of disabled children, compared to one fifth of non-disabled children, lived with a parent/s with a DDA-defined disability. A further 1.5% of disabled children and 7% of non-disabled children lived within a household with one or more adults, who was not a parent, but who also had a DDA-defined disability.

**Table 3:** Living circumstances of disabled children compared to non-disabled children, FRS, 2004/5

	Child has DDA-disabled		No DDA disability		
	***n***	***%***	***n***	***%***	

Lone parent family	406	34.1	3797	25.6	X^2^* = 414.6, <0.0001
Two adult family	783	65.9	11026	74.4	

Median number of children in household	2.00	--	2.00	--	z = -0.595**, NS

Lives with 1 or more siblings with a DDA-defined disability	293	24.6	1078	7.3	X^2^*** = 5412.2, <0.0001

1 or more adults with DDA disability in family unit	543	45.7	2877	20.1	X^2^* = 418.6, <0.0001

1 or more adults with DDA disability in household	560	47.1	3214	21.7	X^2^* = 393.3, <0.0001

Housing tenure:					
*Rented/other*	563	47.4	4935	33.3	X^2^* = 95.9, <0.001
*Owner-occupied*	626	52.6	9888	66.9	

Median number rooms house	5.00	--	6.00	--	z = -5.324**, <0.0001

Live in flat	109	9.2	1298	8.8	X^2^* = 1.2, NS

Live in detached house	223	18.8	3706	25.0	X^2^* = 25.5, <0.0001

Median equivalised total weekly income after housing costs:					
*All*	€334	--	€384	--	z = -6.484**, <0.0001
*1 adult in family*	€277	--	€272	--	z = -0.780**, NS
*2 or more adults in family*	€395	--	€441	--	z = -5.639**, <0.0001
*1 child*	€370	--	€457	--	z = -4.636**, <0.0001
*2 or more children*	€321	--	€365	--	z = -5.006**, <0.0001
Reported ethnicity if head of family:					
*White UK/other*	€344	--	€396	--	z = -6.708**, <0.0001
*Black/minority ethnic/other*	€253	--	€298	--	z = -1.860**, NS

Household income quintiles:					
*Quintile 1*	277	8.6	2942	91.4	X^2^**** = 41.79, <0.0001
*Quintile 2*	280	8.7	2937	91.3	
*Quintile 3*	267	8.4	2912	91.6	
*Quintile 4*	204	8.4	2994	93.6	
*Quintile 5*	161	5.0	3038	95.0	

Reported test statistics;

*Yates continuity correction

** Mann-Whitney U

*** Pearson Chi-square

**** Chi-square for linear trend

Disabled children were more likely to live in rented accommodation than other children. Although there were no statistically significant differences in housing type (house, flats etc.) they were more likely to live in homes with fewer rooms than non-disabled children (Table 3).

### Income, deprivation and debt

The prevalence of DDA-defined disability among UK children appeared to increase across income quintiles, with the highest prevalence of childhood disability found among those in the poorest income quintile (Table 3). Table 3 reports on median equivalised total weekly household income (after housing costs). As a group, disabled children lived in households with lower median incomes than non-disabled children. Although the median income of disabled children living in lone parent households was lower than that for those with two adults, it was similar to that for households with no disabled children in lone-parent households. The households of disabled children from black, minority ethnic/other groups had particularly low median incomes.

Table 4 examines the proportions reporting they wanted or needed, but could not afford, specific items generally considered important for families to have. It shows that on almost every measure, families with disabled children were more likely than other families to report not being able to afford items and activities they wanted or needed. The median total deprivation score for families with disabled children (2.00) was higher than that for other families (1.00) (Mann-Whitney U, z = -8.690, p < 0.0001). This suggests that disabled children and their households experienced greater deprivation than other households with children.

**Table 4:** Social and material deprivation: items perceived as needed or wanted but which can't afford, FRS, 2004/5

Item parent perceived as needed or wanted but which can't afford	Children with DDA disability		Non-disabled children		
	**N**	**%**	**n**	**%**	**p**

**Child specific deprivation**					

Family holiday away from home for 1 week a year	461	38.9	4741	32.1	X^2 ^= 22.9, p < 0.0001

Enough bedrooms for every child of 10 or over of different sex to have own bedroom*	49	22	476	18.5	X^2 ^= 1.4, NS

Leisure equipment such as bicycle	135	11.4	1219	8.3	X^2 ^= 13.6, <0.0001

Celebrations on special occasions -- birthdays, Christmas or other religious festivals	71	6	680	4.6	X^2 ^= 4.4, 0.036

Go swimming at least once a month	161	13.6	1522	10.3	X^2 ^= 12.3, <0.0001

Do a hobby or leisure activity	114	9.6	1042	7.1	X^2 ^= 10.4, 0.001

Have friend round for tea or snack once a fortnight	143	12.1	1224	8.3	X^2 ^= 19.6, <0.0001

Go to toddler group/nursery/playgroup at least once a week	26	7.6	402	6.7	X^2 ^= 0.3, NS

Go on school trips	96	8.6	812	6.5	X^2 ^= 7.5, 0.006

Have an outdoor space or facilities nearby where can play	269	22.7	2342	15.9	X^2 ^= 37.1, <0.0001

**Household deprivation**					

Enough money to keep home in decent decoration	305	25.8	2759	18.7	X^2 ^= 35.1 <0.0001

Enough money for household contents insurance	293	24.8	2683	18.2	X^2 ^= 31.1 <0.0001

Enough money for regular savings of & pounds10 per money	611	51.6	6013	40.7	X^2 ^= 53.3 <0.0001

Enough money for 2 pairs of shoes for each child	209	17.7	1516	10.3	X^2 ^= 61.4 <0.0001

Enough money to replace worn out furniture	452	38.2	4591	31.1	X^2 ^= 25.3 <0.0001

Enough money to replace or repair major electrical goods	302	25.5	3350	22.7	X^2 ^= 4.8 0.028

NS = Not statistically significant

* question asked if two or more children aged 10 or over of opposite sex in household

Table 5 shows the proportions reporting falling behind with particular payments. Debt was more common in families with disabled children. The highest proportion reporting debts was found among families with both disabled children and disabled adults. Being behind with payments for council tax, water rates and telephone bills were the most commonly reported sources of debt.

**Table 5:** Proportions in families who report falling behind with payments, FRS, 2004/5

	Children with DDA disability		Non-disabled children		
**Areas where behind with payments**	**n**	**%**	**n**	**%**	

Electricity payments	84	7.1	571	3.9	X^2 ^= 28.3; <0.0001

Gas payments	89	7.5	551	3.7	X^2 ^= 39.9; <0.0001

Other fuel payment	8	0.7	114	0.8	X^2 ^= 0.04; NS

Council tax	125	10.6	957	6.5	X^2 ^= 28.3; <0.0001

Insurance policies	7	0.6	47	0.3	X^2 ^= 1.7; NS

Telephone bill	105	8.9	731	5.0	X^2 ^= 33.2; <0.0001

TV/video payments	24	2.0	177	1.2	X^2 ^= 5.4; NS

Other HP payments	58	4.9	342	2.3	X^2 ^= 28.9; <0.0001

Water rates	116	9.8	815	5.5	X^2 ^= 35.8; <0.0001

**1 or more debts**	313	26.5	2393	16.2	X^2 ^= 81.0; <0.0001

NS = Not statistically significant

To examine whether differences in social circumstances evident between disabled and non-disabled children in bivariate analyses could be explained by variations in demographic or household circumstances, multivariate logistic regression analyses were carried out (see Table 6). The age, sex and ethnicity of children continued to be associated with childhood disability when other factors were controlled for, with older children, boys and children from white ethnic groups having greater odds of having a DDA-defined disability than younger children, girls or those from black and minority ethnic groups. The association between living in a lone-parent family and childhood disability remained evident at the multivariate level. For disabled children the odds of living in a lone-parent household were 26% greater and of living with a parent with a DDA-defined disability over three times greater than for non-disabled children after adjustment for confounding variables. Housing tenure remained associated with childhood disability, with the odds of living in rented accommodation being 49% greater for disabled children than non-disabled children.

**Table 6:** Logistic regression analyses, FRS 2004/5

	DDA disability			
	**Bivariate**		**Multivariate**	

	***Odds ratio*** ***(95% confidence intervals)***	***p***	***Odds ratio*** ***(95% confidence intervals)***	***p***

**Age of child (years)**				
0-4	1.00			
5-11	2.15 (1.80, 2.57)	<0.0001	2.07 (1.72, 2.48)	<0.0001
12-15	2.65 (2.20, 3.20)		2.39 (1.97, 2.89)	<0.0001
16-18	2.21 (1.74, 2.80)	<0.0001	2.07 (1.62, 2.65)	<0.0001

**Sex of child**				
Girl	1.00			
Boy	1.58 (1.40, 1.78)	<0.0001	1.59 (1.40, 1.80)	<0.0001

**Ethnic group**				
Black/ethnic minority/other	1.00			
White UK/other	1.43 (1.15, 1.78)	0.001	1.47 (1.17, 1.84)	0.001

**No. of adults in family**				
2 adults	1.00			
1 adult	1.51 (1.33, 1.71)	<0.0001	1.26 (1.09, 1.45)	0.002

**No. of dependent children in family**				
2 or less	1.00			
3 or more	1.10 (0,97, 1.25)	0.131	0.93 (0.81, 1.08)	0.932*

**No. of adults with DDA disability/LLSI**				
None	1.00			
1 or more	3.36 (2.96, 3.76)	<0.0001	3.04 (2.68, 3.45)	<0.0001

**Housing tenure**				
Owner occupied	1.00			
Rented/other	1.80 (1.60, 2.03)	<0.0001	1.49 (1.30, 1.71)	<0.0001

* Not statistically significant

## Discussion

This paper has provided estimates of the number of children in the UK defined as disabled according to the DDA and described the circumstances of this group and their households. Before conclusions are drawn, it is important to note several features of the study.

A strength of this study is that it uses data from the FRS, a nationally representative cross-sectional survey with a high response rate and data on a relatively large number of children and young people age 0-18. The overall prevalence estimates generated therefore, are likely to be reliable and valid. As the prevalence of child disability in the UK is relatively low, however, the size of some sub-groups of disabled children was small. In total there were only 90 disabled children from black, minority ethnic or mixed parentage groups. As a result, it was only possible to provide analyses for white UK/other and black/minority ethnic/other groups rather than by individual ethnic group. It is acknowledged that such analyses are limited and fail to provide sufficient information on disabled children from particular ethnic backgrounds. To generate accurate national and local prevalence estimates and data on the circumstances of different ethnic groups in the UK, a substantially larger and/or boost sample is required.

A further strength of the study is both its use of measures of income poverty and of material deprivation. Income measures alone are not necessarily good measures of living standards, particularly at the bottom of the income distribution [21,22]. In using measures of income, lack of socially perceived necessities and debt, this study encompasses a broader range of measures of living standards.

The findings reported here provide up-to-date data on the numbers and circumstances of a nationally representative sample of disabled children and their households in the UK. Using a definition of disability enshrined in the DDA, 952, 741 children (7.3%) in the UK in 2004-5 were reported to be disabled. The overall prevalence estimate of child disability reported in this study is higher than those reported from the FRS for earlier years and for the estimate for the same FRS survey year published by the Department for Work and Pensions. This is explained by use of different definitions of disability across surveys and across years within the same survey. The most recent DDA-related definition of disability was used to generate these analyses. Using this more inclusive measure increased the prevalence estimate by almost two percentage points and 250,000 children above the published estimates for 2003/4. This illustrates how changes to disability definitions and survey question sets within surveys can affect prevalence estimates.

Other recent UK surveys and data sources have produced estimates ranging from 4.5% to 16% [23], thus the prevalence estimate generated by our study falls mid-range. Mooney *et al *[24], using published figures and information from local authorities have suggested that in England the mean percentage of disabled children was lower, falling between 3% and 5.4%. This lower range estimate may be attributable, at least in part, to the definitions of disability employed and the populations of children included in the data sources, whose primary purpose may not be to capture the whole population of disabled children. Furthermore, prevalence estimates derived from sources relating to private households, as in this study, will not collect data on the small number of children living elsewhere, for example in residential establishments. As a result, they are likely to underestimate the prevalence of childhood disability. This suggests that data users need to understand and be clear about how the estimates they use are derived and population coverage.

The significant association between the age of the child and disability found in this study is consistent with other research [25]. Lower prevalence among younger children is likely to be explained in part, by the fact that a range of conditions do not manifest themselves until later, and some become progressively more activity limiting as the child gets older. Failure by health, education and social care agencies to identify disability early in a child's life however, may also play a part in some cases.

Our findings indicate that disabled children and their households live in different personal circumstances, and substantially more disadvantaged material circumstances than their non-disabled children. Gordon *et al's *(2000) reanalysis of the Office of Population, Censuses and Surveys (OPCS) disability survey also highlighted the poverty and poor living standards of disabled children in Britain in the 1980s [6]. The analyses presented here suggest that little has changed. Disabled children in the UK today continue to experience income inequality and material and social disadvantage. While an association between poverty and childhood disability is well-established [26], little is known about the precise nature of the relationship between childhood disability and social disadvantage or the extent to which factors such as low income precede or follow impairment. It was clear however, that the household incomes of disabled children and their families were, on average, lower than those of non-disabled children and that they experienced higher levels of debt and social deprivation. This is likely to be attributable to a number of factors. Households with disability children have a greater dependence on social security benefits and are faced with the additional financial costs associated with caring for a disabled child [27-30]. It has been estimated that in the UK, families need incomes that are 10% - 18% higher than similar families with non-disabled children to have the same living standard [31]. Higher levels of low income, debt and social deprivation are likely to be linked to the higher prevalence of lone-parenthood and parental disability in households with disabled children [32,33].

This study highlighted that one third of disabled children lived in lone parent households, a similar proportion to that reported by Emerson and Hatton, 2007 [30] but a considerably higher proportion than that identified by the OPCS disability survey, which reported that 19% of disabled children lived with a lone parent [6,34]. The association between childhood disability and lone parenthood persisted when social disadvantage and other factors were controlled for.

An association between lone parenthood and childhood disability has been reported elsewhere in the UK [35,36] and in other countries, including the United States [37,38]. Reasons for this observed relationship between lone parenthood and disability are however, unclear and further research is needed. Higher divorce rates among parents of disabled children, lower rates of repartnering and a higher prevalence of births of disabled children to lone mothers are possible explanations. While studies, in general, have found higher divorce rates among parents of disabled children than non-disabled children, some studies suggest that this is not universally the case, with divorce rates among parents of children with Downs syndrome occurring proportionally less often than among other parents [39]. While further research is need to explain the observed relationship between of lone parenthood and disability, given the further association of these with poverty and material deprivation, and evidence of high parental workloads linked with caring for some disabled children, the difficulties faced by these families need to be addressed through services and public policy.

Our findings indicate that disabled children in the UK are more likely than non-disabled children to live in rented accommodation. This confirms the findings from other research [30,40]. Other studies indicated lack of space, and poor access inside and outside the home as commonly reported problems for families with disabled children [41,40,25]. Together these data underline the need for national and local policies and services that seek to address the housing needs of disabled children and their households.

A significant finding of this paper is evidence of a clustering of child and adult disability. First, disabled children were more likely to live with disabled siblings and other disabled children than non-disabled children. One quarter of children with a DDA defined disability lived with one or more siblings who also had a DDA-defined disability. To date, information on the number of UK disabled children in any one family unit has been unclear. The OPCS survey did not collect data on the number of disabled children living in a family or household. Gordon *et al *(2000) have suggested that estimates from other studies for the number of families caring for two or more disabled children range between 4% and 11%. A number of these studies however were based on data from families who had made successful applications to the Family Fund Trust. Such studies are unlikely to be representative of the wider population of disabled children because not all families of disabled children apply for assistance from the Trust and in addition, its eligibility criteria preclude access by middle and higher income families [6]. Second, we found a relatively high prevalence of parental disability among parents of disabled children. The association between child and adult disability persisted when social disadvantage and other factors were controlled for. Although an association between child and adult disability in the same household has been described [42], the research did not control for the impact of confounding factors, as we did. In addition, the proportion of disabled children living with disabled adults was found to be smaller than was evident in our study.

Given the hereditary nature of a small number of impairments and health conditions and the relationship between poverty, caring and disability for adults and children, evidence of disabled children living with disabled parents should not be surprising. Further research is required to establish whether parental disability precedes or follows the experience of parenting a disabled child.

## Conclusion

While there is a rich seam of data from qualitative studies on the experiences of disabled children and their households, detailed and reliable quantitative data on the prevalence of childhood disability and the characteristics and circumstances of this important group have been noticeably lacking in recent years. The analyses presented here provide useful estimates of the proportions of disabled children living in particular circumstances in the UK and offer an overview of how their circumstances compare to non-disabled children. Our findings highlight the need for further research on larger data sets to generate more precise prevalence estimates for childhood disability by ethnic group, and further work to investigate the associations between childhood disability and lone-parenthood and parental disability. Given the relationship between positive health, social and education outcomes and poverty and material deprivation, improving the circumstances of disabled children is likely to be crucial.

## Abbreviations

DDA: Disability Discrimination Act; FRS: Family Resources Survey; UK: United Kingdom; OPCS: Office of Population, Censuses and Surveys.

## Competing interests

The authors declare that they have no competing interests.

## Authors' contributions

All authors conceived of the study and contributed to the study design. CB carried out the data analysis and wrote the paper. JR and NS contributed to the editing of the paper. All authors read and approved the final manuscript.

## Pre-publication history

The pre-publication history for this paper can be accessed here:

http://www.biomedcentral.com/1471-2431/10/21/prepub
